# Investigating the impact of user perception and gamification elements on repurchase decisions in game live broadcasting

**DOI:** 10.3389/fpsyg.2025.1492832

**Published:** 2025-01-22

**Authors:** Guihua Zhang, Junwei Cao, Mengli Huang, Shang Meng

**Affiliations:** ^1^School of business, Xinyang Normal University, Xinyang, China; ^2^Dabie Mountain Economic and Social Development Center, Xinyang, China; ^3^School of business, Yangzhou University, Yangzhou, China; ^4^Anyang Institute of Technology, Anyang, China

**Keywords:** live business, game live viewers, A-P-B, affordance, repurchase

## Abstract

As a branch of online live broadcasting, the development of game live broadcasting is also getting faster and faster. Based on the theory of affordance-psychological outcome-behavioral (A-P-B), this paper introduces three gamification elements of user activity visibility, user level visibility and purchase effect visibility, and three user-perceived experience values of perceived hedonic value, perceived cognitive value and perceived social value to establish the model. After collecting data through questionnaires, an empirical analysis based on partial least squares structural equation modelling (PLS-SEM) was carried out using SPSS and SmartPLS data analysis methods to explore the influencing factors of viewers’ re-purchase behaviors in game live broadcasting from the perspective of the visibility of gamification elements. The results of this study show that in game live broadcasting, there is a certain influence between the visibility of gamification elements and the perceived value of users, and that the visibility of user activity, the visibility of purchasing effects, and the visibility of user level all have a significant positive effect on the perceived value of the user experience. This study enriches the literature on the business aspects of game live broadcasting and consumer behavior and has a certain significance in guiding live broadcasting business scenarios to improve the re-purchasing behavior of live broadcast viewers.

## 1 Introduction

Online game broadcasting is a kind of real-time video social media that combines traditional broadcasting and online gaming. In recent years, with the emergence of various live streaming platforms such as Twitch, YouTube, Douyu, and Hutong, the online live streaming industry has been developing rapidly. China’s live broadcasting industry started late but is developing rapidly, and the industry has entered a mature stage of development. A study pointed out that since 2017, the market scale and user scale of China’s game live broadcasting industry have shown rapid growth, with the market scale reaching 30 billion yuan in 2020, an increase of 44% over the previous year; the number of game live broadcasting users reached 350 million, an increase of 15% year on year (Cunningham et al., 2019). At the same time, the business model of the game live broadcasting platform in China is constantly being enriched, the overseas market is constantly expanding, and the whole platform is more diversified and globalized. According to the statistical report of Avery Consulting Research Institute, the market scale of China’s game live broadcasting will reach 53 billion yuan in 2022, an increase of 21.8% compared with 2021, the market growth rate is beginning to slow down but still maintain a certain degree of growth, and the industry has entered an orderly competitive environment.

Although the development of China’s game live broadcasting industry is levelling off, there are still various problems. In general, there are serious similarities in the content of game live broadcasts, defective platforms with incomplete functions, various disputes over copyright issues, and so on, this has a certain impact on the re-purchase behavior of game live viewers (Zhang and Liu, 2024). A study found that most of the game live content is too homogeneous, some focus on packaging and advertising, and these behaviors have a direct impact on the audience’s live viewing experience, which in turn has led to a serious decline in the number of re-purchases of live game users (Matusov, 2020). Understanding the factors that influence the re-purchase of live gaming viewers is important because the online live gaming industry is growing rapidly and viewer purchase behavior on live streams plays a key role in generating revenue for companies. Users repurchase behavior is extremely important for gaming companies and live streaming platforms. Previous research on service marketing states that managing existing customers is more effective than acquiring new ones (Guerola-Navarro et al., 2024). Therefore, understanding the re-purchase behavior of existing viewers of games live is important to improve the sustainability of platforms and the profitability of game providers.

Many scholars have studied the factors affecting repurchase behavior in the game live industry. These factors include game content affordance, information affordance, perceived experiential value. Factors such as game content-related elements, information affordance and platform functionality can increase audience engagement, trust and satisfaction with live streaming services, which influences the emergence of re-purchase behavior. Su et al. (2020) suggest that affordances such as in-game props in live streaming can sustainably stimulate users’ willingness to buy. These elements can capture viewers’ attention and interest, increase their engagement during live streaming, and create a sense of immersion in the game world. Cao et al. (2022) proposed that verbal intimacy and virtual physical intimacy of anchors positively influence users’ continued purchase of expensive virtual gifts. Meanwhile Kaur et al. (2023) suggests that perceived enjoyment is an important prerequisite for determining a consumer’s intention to engage in gamification. The summary found that factors such as game content-related elements, information provision, and platform functionality can increase viewer engagement, trust, and satisfaction with live streaming services, thereby influencing the emergence of repurchase behavior.

In live gaming, the availability of various information including live rules, anchor background, live schedule and other information plays a crucial role in viewers’ repurchase behavior. Several studies have shown a potential correlation between information availability and gamification elements. For example, providing clear and easy-to-understand information about live games can help viewers understand and engage with the content, while gamification elements such as viewer points and badges in live broadcasts can make them more interesting and motivating. Some scholars have pointed out that by increasing the visibility of gamification elements, live streamers can achieve higher audience engagement and satisfaction with the content (Qian et al., 2023), and that user engagement and satisfaction are often closely related to users’ purchasing behavior. Therefore, based on the above research background, this paper poses the following research question: how does the visibility of gamification elements affect viewers repurchase behavior? This study aims to analyze the audience’s repurchase behavior in live game broadcasting from the perspective of gamification elements, starting from the aspects of information visibility and perceived experience value.

## 2 Literature review and theoretical background

### 2.1 Affordance-psychological outcomes-behavior theory

The A-P-B theory is a theoretical model widely used in the fields of psychology, marketing and advertising to describe the process of generating and changing human behavioral A-P-B represents three stages: affordance (A), psychological outcome (P) and behavior (B). This model can help people understand why and how behaviors change (Tillas et al., 2017), so that effective strategies can be better developed and implemented. The affordance (A) stage refers to the stimulus or event that triggers a change in behavior, which can be an external factor such as advertising, publicity, social media, etc., or an internal factor such as a perceived need, pressure, or desire (Xu et al., 2022). At this stage, attention is drawn and relevant information is perceived to elicit further responses. The psychological outcome (P) stage refers to people’s reactions, such as emotions, attitudes, and beliefs, to a triggering event or stimulus. This response can be positive, such as interest, like or approval, or negative, such as dissatisfaction, rejection or disgust (Karahanna et al., 2018). These psychological effects can influence people’s decisions and behaviors so that they are more likely to take a particular action. The Behavioral (B) stage refers to the actual actions that people take based on their psychological effects, such as buying goods, participating in activities or following certain rules (Xu et al., 2022). In this stage, people take action to fulfil their needs, desires or goals.

A-P-B theory highlights the complexity and diversity of human behavioral change and provides a systematic approach to understanding why people take certain actions and how they are motivated to take particular actions (Xu et al., 2022). In practice, this model can be used to design and implement marketing strategies, change consumer behavior, improve employee performance and much more. The concept of affordance is important in consumer behavior because it helps explain how consumers perceive and respond to products and environmental. Su et al. (2020) have suggested in their study that in the field of webcasting, the online affordance of webcasting leads to psychological outcomes for consumers, such as social presence and self-esteem, and a positive experience further leads to behavioral outcomes, i.e., purchases. Understanding affordances and their impact on psychological outcomes is of interest and research for designing effective products and environments that can positively influence consumer behavior.

### 2.2 Gamification elements

Deterding et al. (2011) state that gamification is “the use of game design elements in non-game environments and this definition identifies four key points for understanding gamification: the game, the elements, the design and the non-game environment”. Huotari and Hamari (2012) in contrast, emphasize that gamification is the process of making all kinds of activities more like games. His definition of gamification places more emphasis on the dynamic process of shifting from non-gamification to gamification. His study argues that gamification is not simply inserting gamification elements into activities, but rather selecting appropriate gamification elements based on the characteristics of the activity and applying the elements to the activity in order to achieve integration of the elements with the activity. A study by Groening and Binnewies (2019) breaks down the three categories of gamification elements, noting that socially oriented social elements include, in particular, co-operation, competition, collective participation, and interaction; achievement-based elements include, but are not limited to, challenges, points, leaderboards, game logos, and levels; and immersive elements include, primarily, in-game avatars, in-game awards ceremonies, and virtual personalities.

In the past, a great deal of research on gamification has focused on areas such as education, health, and management. With the development of smartphones and interactive technologies, games are becoming increasingly important and fascinating as an educational resource in a variety of educational settings. Many studies have shown that in game-based learning, teachers achieve their educational goals by leading students to play games together, and that “play” often plays a crucial role in the learning process (Putz et al., 2020; Saleem et al., 2022). In the field of management studies, many scholars believe that gamification “is an effective way to increase the motivation and engagement of employees, customers, patients and students as well as other stakeholders” (Wolf et al., 2020). In health management, gamification design has become the main means for health management platforms to motivate individuals, providing new ideas for the future development and growth of health management platforms. Scholars believe that the gamification design of health management platforms can stimulate individuals’ interest in health management and motivate them to participate in their own health management (Wang et al., 2021).

In the process of live webcasting, the use of gamification design allows the platform to go about establishing unique advantages in a more interesting and distinctive way, which not only enhances the user’s sense of experience, but also increases the user’s engagement behavior. Gamification design can increase the degree of consumers’ perceived experience and enable consumers to derive a higher degree of fun from it, and live streaming platforms with gamification design are more likely to engage consumers in transactions compared to online platforms without gamification design. A study by Tobon et al. (2020) found that visual rewards, including trophies, badges and collectible cards, and gamified themes were key factors influencing user retention and platform engagement. This suggests that a gamified design with visual gamification elements can increase user engagement and experience during webcasting, thereby increasing user activity. Meanwhile Chang and Yu (2023) advocate that gamification design during live broadcasting can stimulate users’ continuous pursuit of pleasurable experiences and positive behaviors, and he argues that if certain gamification elements are added to the live broadcasting process, it can increase users’ positivity and enjoyment, and stimulate their motivation to consume and thus inspire their purchasing behaviors.

### 2.3 Perceived value

Perceived value is a concept originally developed in the field of marketing and refers to the subjective feelings that an individual has when he or she does something. Customer perceived value is “what customers expect from a product or service” and “the ratio of perceived benefit to perceived price” (Boksberger and Melsen, 2011). Customers are ecstatic and expect to get value for their money, in simple terms perceived value can also be interpreted as a subjective evaluation of the purchase behavior of the consumer when there is a purchase behavior that weighs up the gains and losses in their mind. Watanabe et al. (2020) found that consumer perceived value consists of five dimensions: health value, safety value, social value, hedonistic value, and environmental value based on users’ willingness to purchase organic agricultural products, and Hsin Chang and Wang (2011) proposed a multifactor theory of perceived value from the perspective of consumer behavioral patterns. He argued that perceived value should include social value, functional value, cognitive value, emotional value and situational value.

Delivering superior customer value to achieve customer satisfaction is critical to gaining a competitive advantage, so too in the live webcasting space, user-perceived value plays a critical role. Lim et al. (2020) study examined how emotional identification and prosocial relationships influence users’ repeated viewing in a live game show with a prosocial cognition theory perspective, and the findings found that viewers’ emotional engagement and identification significantly influenced viewer loyalty. Singh et al. (2021) assessing determinants influencing continued use of live streaming services extended the perceived value theory to include innovativeness and enjoyment, the study found that perceived hedonism is a key factor leading to users continued viewing of live streams. The study found that perceived hedonism is a key contributing factor to users continued use of live streaming services. And Meng and Lin (2023) and others suggest that in the process of webcasting, the anchor popularity, the degree of interaction between the anchor and the user audience and the related special services have a certain positive impact on consumer purchasing behaviors.

By combing through this literature one can see that Perceived Hedonic Value is the enjoyment or pleasure that a user derives from an experience or activity, such as entertainment, fun, or excitement. Perceived Cognitive Value is the practical or informational benefit that users derive from an experience, such as learning, understanding, or gaining insights, and Perceived Social Value is the sense of social connectedness or belonging that users experience through an activity, often driven by interactions with others. In a live streaming environment, this could include interacting with a moderator, participating in a chat discussion or feeling part of a community. In this case, the perceived value plays an intermediary role, because in webcasting, both the anchor’s interaction and the platform’s special features will bring different perceived experiences to the audience users, which will increase the user’s participation in the live broadcast, and then realize the purchasing behavior.

## 3 Research model and questionnaire

This study adopts the Affordance-Psychological Outcome-Behavior (A-P-B) theory, focusing on one of the effects of gamification elements on users’ re-purchase behaviors from the affordance perspective of gamification elements in the field of game live broadcasting in webcasting. Between the research background of the game live broadcast, not all gamification elements can be applied, so combined with the development of the live broadcast of online games in the current situation of this paper will be used to analyses the affordance of three gamification elements: user activity, user level and purchase of special effects Between the research background of the game live broadcast, not all gamification elements can be applied, so combined with the development of the live broadcast of online games in the current situation of this paper will be used to analyses the affordance of three gamification elements: user activity, user level and purchase of special effects in the current situation of the live broadcast of online games in the current situation of the live broadcast of online games in the current situation of the live broadcast of online games in the future. The three gamification element affordances of user activity, user level and purchase of special effects will be analyzed and studied. Psychological outcomes are studied using perceived value, as perceived value explains both the purchasing behaviors of users and is one of the key factors influencing user engagement and satisfaction psychological outcomes are studied using perceived value, as perceived value explains both the purchasing behaviors of users and is one of the key factors influencing user engagement and satisfaction.

**Figure 1:**
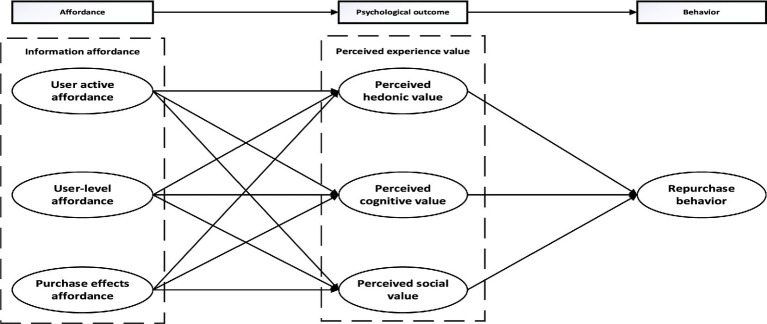
Conceptual model of factors influencing the re-purchase behavior of game live viewers.

Based on previous research, this study constructs a research model between gamification element revelation, perceived experiential value and user repurchase behavior from the perspective of gamification revelation under the theoretical framework of burden psychological outcome behavior. Combined with previous studies, this study subdivided the informational burden into user activity burden, user level burden, and purchase special effects burden, and subdivided the perceived value into three variables, namely, perceived hedonic value, perceived cognitive value, and perceived social value, to jointly explore their effects on user repurchase behavior (see Figure 1).

### 3.1 Hypothesis development

#### 3.1.1 Hypothesis of the relationship between gamification element affordances and perceived experiential value

Appropriate use of gamification elements can improve user motivation and engagement (Suh et al., 2018), and the incorporation of visual gamification elements, such as visual trophies, badges, and other symbols, into designs can improve user experience and engagement. This also implies that adding visual gamification elements (e.g., visible levels, active markers, or special effects rewards) to the design of live gaming platforms is effective. By adding active gamification elements to live gaming streams, live stream viewers can complete tasks set by the gamification elements to earn active markers or rewards, which increases viewers’ motivation to watch live streams, and completing the tasks results in a more interesting and engaging experience.

Based on the gamification and affordance perspectives, user activity visibility refers to the ability of a system or platform to clearly demonstrate a user’s level of activity or engagement on the platform through design elements. It enables users to intuitively perceive the frequency and depth of their own or others’ interactions in the system, e.g., through indicators such as the number of visits, task completion rate, and participation time to reflect user activity. When live viewers are gradually active, they are also more able to experience the service functions of the live broadcast platform as well as feel the live content of the anchor, deepening their awareness of the game live broadcast. Chen et al. (2016) had researched and concluded that in games, social interactions can significantly increase players’ enjoyment and engagement. By interacting with other players, players are more likely to feel satisfied and enjoy the game experience. Burke et al. (2011) have suggested in their study that there is a relationship between social capital and its activity level. In game live broadcasting, the higher the user’s activity level, the more it can increase its exposure, which can attract the attention of the anchor and other fans and improve the possibility of becoming friends with other users, which is also a reflection of the user’s perception of social value. Accordingly, the hypothesis of this study was formulated:

H1a: User activity affordance positively affects perceived hedonic value.

H1b: User activity affordance positively affects perceived cognitive value.

H1c: User activity affordance positively affects perceived social value.

Based on the gamification and affordance perspective, user level visibility is the ability to clearly display a user’s current level or status through design elements in a system or platform. It enables users to visualize their rank status and relative position to other users, for example, by presenting information about the user’s rank in the form of badges, icons, rank names, or progress bars. According to Yu and Huang (2022) by adding a gamification element that embodies rank in game live broadcasting, live viewers will gain a higher user rank identification through the length of time spent watching game live broadcasts, doing tasks, and offering rewards. When enjoying this gamification service, live viewers will be able to experience the fun of game live broadcasting and perceive a stronger hedonic value. A higher user level usually indicates a longer online time and experience of watching live broadcasts, so users can have a more in-depth experience of live game broadcasts. At the same time, higher rank markers will attract the attention of other lower ranked fans in the live stream as well as more popularity from the game hosts, and higher ranked users will be more vocal and present, which will probably increase users’ perception of social value (Sajjadi et al., 2019). Accordingly, this study proposes the hypothesis:

H2a: User level affordance positively affects perceived hedonic value.

H2b: User level affordance positively affects perceived cognitive value.

H3c: User level affordance positively affects perceived social value.

Based on the gamification and affordance perspective, the visibility of user-purchased special effects refers to the ability of a platform or system to display virtual special effects or exclusive decorations purchased by users through design elements. It enables users and others to visualize the presence of these effects, such as avatar boxes, chat bubbles, dynamic animations, etc., thus enhancing the user’s sense of identity and interactive experience. By combing through the literature of Kuo and Chuang, (2016), it can be learnt that adding appropriate gamification elements to games can increase the possibility of users’ consumption, and the direct inclusion of the availability of purchasing special effects in game live streaming will further stimulate consumers’ purchasing behavior. Users watching the live broadcast generate consumption when watching the live broadcast, presenting gorgeous special effects can increase the user’s entertainment experience and enhance the hedonic value of the user, while the design of the special effects also allows users to differentiate it from other platforms and have a deeper knowledge of the live broadcast platform. Increasing the availability of purchasing special effects increases the exposure of the user’s account to a greater extent, and the appearance of special effects during gift-giving and rewarding will even attract the attention of the anchor as well as other users (Wang et al., 2024), which will increase the likelihood of the user’s perception of social value. Accordingly, this study proposes the hypothesis:

H3a: Purchasing special effects affordance positively affects perceived hedonic value.

H3b: Purchasing special effects affordance positively affects perceived cognitive value.

H3c: Purchasing special effects affordance positively affects perceived social value.

#### 3.1.2 Hypothesis on the relationship between perceived experiential value and viewer repurchase behavior

Users’ engagement to a particular platform must be based on their own needs to start with, and purchase intention is related to subjective judgement and consumer surveys and based on the consumer’s desire to purchase certain products and services (Alavi et al., 2016; Juster, 1966). When consumers have a strong intention to buy, the probability of successful consumption is greater and the likelihood of the transaction being concluded is higher. Burke et al. (2011) suggest that consumer intention is the probability of making a final decision about a particular product under the influence of a number of factors and is an important predictor of consumer behaviors. Willingness to buy refers to how strong a consumer is about having the desire and desire to buy a certain commodity. According to Chen and Lin (2018) and others, perceived value will have a certain impact on viewers’ purchasing behavior in live streaming services, and perceived hedonic value will provide a mediating role. Watching the process of game live broadcasting users from spending a certain amount of time and energy, from the game live broadcasting to obtain a pleasant mood, the display of the host’s superb technology, and the performance of rich live content. With the depth of the perceived value dimension, the purely direct benefit gain can be gradually expanded to similar emotional, cognitive and other value dimensions (Bettiga and Lamberti, 2017). Accordingly, this study proposes the hypothesis:

H4a: Perceived hedonic value positively influences viewer re-purchase behavior.

H4b: Perceived cognitive value positively influences viewers' re-purchase behavior.

H4c: Perceived social value positively influences viewers' re-purchase behavior.

### 3.2 Questionnaire survey

In this study, questionnaires were designed to collect data, which were then analysed to empirically test the proposed theoretical relationships. In order to ensure the quality of the scales and the validity of the questionnaire, all the items measured in this study were based on well-established scales from other studies, adapted to the themes and needs of this study. For the scale of gamification element affordances, this study refers to the study by Su et al. (2020). The scale was divided into three aspects: user activity affordance, user level affordance, and purchase of special effects affordance, and each measure was adapted accordingly based on the real-world context and the needs of the study. For the Perceived Experiential Value Scale, this study refers to the study by Su et al. (2020) and also to the study by Kim et al. (2012). The scale is divided into three dimensions: perceived hedonic value, perceived cognitive value, and perceived social value, and each measurement item is adjusted accordingly based on the real-world situation and research needs. For the Repurchasing Behavior Scale (RBS), this study refers to the study by Wen et al. (2011) and also to the study by Mittal and Kamakura (2001). Each measurement item was adjusted according to the real situation and the needs of the study.

A five-point Likert scale will be used in this study using a survey with measures ranging from strongly disagree to fully agree for each construct (1 = strongly disagree, 2 = disagree, 3 = fairly, 4 = agree, and 5 = fully agree) In order to improve its validity, the measurements presented in the existing literature have been slightly modified to suit the present study and experts in the field have been invited to review the questionnaire. In addition, this study conducts smaller preliminary tests to assess and enhance the validity and reliability of the questionnaire. Details of the questionnaire form can be found in Appendix. The questionnaire for this study was distributed online through social networks. The following measures were taken in the questionnaire: (1) the questionnaire is anonymous; (2) the content and purpose of the survey will be clearly expressed to the respondents; (3) all participants have the freedom to answer or not to answer the questionnaire; (4) the questionnaire does not involve private information; (5) the data collected from the survey will only be used in this study; and (6) all participants will receive a gift after answering the questionnaire.

During our questionnaire data collection process, we did not collect any identifying information such as respondents’ names or contact details, nor did we collect any biological data. Additionally, respondents did not participate in any form of psychological experiment. Therefore, according to the policies and regulations of our institution’s Institutional Review Board (IRB), this study does not fall within the scope of research requiring ethical review. Specifically, ethical review by the IRB is generally required for research involving sensitive personal data or intervention-based experiments, neither of which apply to our study. As such, there is no need to submit an IRB review application.

Between February 26, 2024 and March 20, 2024, our questionnaire was posted to the eligible live gaming group. To complete this survey, respondents were selected from the major live gaming platforms who had at least one purchase during a live gaming session and used the live streaming platform at least once a week for at least 1 month. We then commissioned these respondents to invite as many of their friends as possible to participate more in the survey and each successful invitation to a valid respondent would receive a small reward. By using this snowballing method for the questionnaire, we received 268 responses by the time the survey closed. Finally, from these 268 responses, the data was screened and deleted according to the survey requirements, resulting in 232 valid questionnaires (86.5%).

## 4 Data analyses and results

### 4.1 Analysis methods

This study first used descriptive statistics to analyze the demographic characteristics of our collected sample and then used structural equation modelling to test the reliability and validity of the measurement hypotheses. Structural equation modelling can be divided into two types, one is covariance-based structural equation modelling (CB-SEM) and the other is partial least squares-based structural equation modelling (PLS-SEM). PLS-SEM structural equation modelling was used in this study. Firstly, the least squares method is useful for prediction and is less restrictive than other sample size methods. Second, Hair et al. (2017) showed that PLS-SEM is more suitable than CB-SEM for measuring complex models, especially models with more than 6 variables. Finally, PLS-SEM allows the analysis of non-normally distributed data. The model for this study had seven variables and was an exploratory study with multivariate normality tests, which indicated that the data did not satisfy a normal distribution (skewness: b = 143.8, *p* < 0.001; Kurtosis: b = 921.8, *p* < 0.001). In conclusion, this study is more appropriate to use Partial Least Squares Structural Equation Modelling (PLS-SEM) for data analysis.

### 4.2 Descriptive analysis

Demographic information was included in the survey. Of the respondents, 127 (54.7%) were male and 105 (45.3%) were female. The main age group of the respondents was between 20–35 years old (N = 123, 53%). Educational attainment was mainly bachelor’s degree (N = 94, 40.5%) and college degree (N = 45, 19.4%). 27.6% of respondents (*n* = 64) had a monthly income amount of less than RMB 2,000, 21.1% (*n* = 49) had a monthly income amount between RMB 2,000–3,999, 20.7% (*n* = 48) had a monthly income amount between RMB 4,000-5,999, 17.2% (*n* = 40) had a monthly income amount between 6,000–8,999, and 13.4% of respondents (*n* = 31) earned more than 9,000 RMB per month. Of the respondents, 40.1% (*n* = 93) spent on live gaming in the number of times style 1–2 times, 24.1% (*n* = 56) spent between 3–5 times on live gaming, 16.4% (*n* = 38) spent between 6–10 times on live gaming, and 19.4% (*n* = 45) spent more than 10 times. In addition, 47.4% (n = 100) spent less than RMB 100 while watching games live, 20.7% (*n* = 48) spent RMB 101–500, 16.8% (*n* = 39) spent RMB 501–1,000 while watching live games, and only 15.1% (*n* = 35) spent live streams spent more than RMB 1,000 (see Table 1).

**Table 1:** Statistical details of respondents.

Items	Options	Frequency	Percentage
Gender	Male	127	54.7
	Female	105	45.3
Age	Under 20	45	19.4
	20–35 years	123	53
	36–50 years	32	13.8
	Over 50	32	13.8
Educational level	Junior high school and below	38	16.4
	High school degree	28	12.1
	Junior college degree	45	19.4
	College degree	94	40.5
	Graduate and above	27	11.6
Incomes	Less than 2,000 RMB	64	27.6
	RMB 2,000–3,999	49	21.1
	RMB 4,000–5,999	48	20.7
	RMB 6,000–8,999	40	17.2
	Over 9,000 RMB	31	13.4
Number of times purchased	1–2 times	93	40.1
	3–5 times	56	24.1
	6–10 times	38	16.4
	Over 10 times	45	19.4
Purchase amount	Less than RMB 100	100	47.4
	RMB 101–500	48	20.7
	RMB 501–1,000	39	16.8
	Over 1,000 RMB	35	15.1

### 4.3 Common method bias

Common method bias (CMB) is a frequent problem in questionnaire surveys. Harman (1976) one-factor analysis has been widely used to estimate the likelihood of methodological bias prevalent in social science research. Podsakoff et al. (2003) and others have suggested that a single factor must be extracted. If the variance is less than 50 per cent, it indicates that the survey data are less affected by common methodological biases. Because Harman’s one-way test results were close to the 50 per cent threshold, this study further applied the test of common method bias in the structural equations applicable to the partial least squares method. Kock (2015) suggested that if a FLL-VIF value greater than 3.3 occurs it is considered to be suggestive of pathological covariance and that the model will likely exist affected by common method bias. In the present study, the tests for full covariance between the structures were all below 3.3. Therefore, considering the results of the one-way test analysis with the analysis of FLL-VIF values tested for common method bias, it can be assumed that common method bias was not a serious issue in this study.

### 4.4 Measurement model

Before analyzing the structural relationships, it is necessary to assess the reliability and validity of the variables. Validity is further divided into Convergent Validity, which indicates the degree of relationship between the element under test and the factor, and Discriminant Validity, which indicates the difference between concepts. Reliability can be measured by Cronbach’s alpha and Composite Reliability. As shown in Table 2, the CR and Cronbach’s alpha of all variables exceeded 0.7, indicating that the reliability of the data in this study was satisfactory. The AVEs of the variables are all greater than 0.5 and the outer loadings are more than 0.7, indicating that the convergent validity of the data in this study is satisfactory (Hair et al., 2017).

**Table 2:** Assessment of reliability and convergent validity.

Constructs	Items	Loading	Mean (STDEV)	Cronbach’s α	CR	AVE	R^2^
Users activity affordance	UAA1	0.845	3.19 (1.133)	0.853	0.911	0.774	–
	UAA2	0.915					
	UAA3	0.878					
User level affordance	ULA1	0.872	3.26 (1.178)	0.883	0.928	0.811	–
	ULA2	0.933					
	ULA3	0.894					
Purchase effects affordance	GEA1	0.890	3.25 (1.172)	0.886	0.929	0.814	–
	GEA2	0.907					
	GEA3	0.909					
Perceived hedonic value	PVE1	0.858	3.15 (0.951)	0.895	0.927	0.760	0.402
	PVE2	0.897					
	PVE3	0.812					
	PVE4	0.917					
Perceived cognitive value	PCV1	0.882	3.33 (0.999)	0.872	0.921	0.796	0.576
	PCV2	0.936					
	PCV3	0.856					
Perceived social value	PSV1	0.897	2.94 (1.042)	0.901	0.938	0.835	0.318
	PSV2	0.936					
	PSV3	0.907					
Repurchase behavior	REB1	0.917	3.12 (1.076)	0.904	0.940	0.839	0.586
	REB2	0.932					
	REB3	0.898					

Discriminant validity was tested in this study by both Fornell and Larcker’s Test and the Heterotrait-Monotrait ratio (HTMT) Test. As shown in Table 3, the square root of AVE for each variable is greater than the correlation with other variables, and the values of HTMT are all lower than the mean value of 0.85. So, the validity of this study’s discriminant validity is in accordance with the requirements.

**Table 3:** Distinctive validity tests for variables.

	UAA	ULA	PEA	PHV	PCV	PSV	REB
Fornell and Larcker’s test							
UAA	0.880						
ULA	0.423	0.900					
PEA	0.313	0.383	0.902				
PHV	0.483	0.482	0.484	0.872			
PCV	0.347	0.592	0.665	0.661	0.892		
PSV	0.470	0.380	0.419	0.626	0.531	0.914	
REB	0.351	0.283	0.383	0.629	0.520	0.731	0.916
Heterotrait-Monotrait ratio (HTMT) test							
UAA							
ULA	0.486						
PEA	0.360	0.430					
PHV	0.555	0.545	0.538				
PCV	0.397	0.669	0.749	0.743			
PSV	0.527	0.419	0.462	0.678	0.589		
REB	0.395	0.316	0.423	0.683	0.584	0.800	

### 4.5 Analysis of structural models

Before measuring the structural model, this study measured the covariance and the VIF values of all the variables were less than 3, so as far as the covariance is concerned, its not a major issue in this study. After ensuring the reliability and validity of the model, this study tested the hypothesis with the structural model. The results of testing the trajectory coefficients and significance of the structural model are shown in Table 4.

**Table 4:** Measurements for the path model.

Hypothesis	*β*	STDEV	T-value	*p*-value	Support
H1a: UAA → PHV	0.285	0.061	4.685	0.000	Yes
H1b: UAA → PCV	0.024	0.050	0.478	0.633	No
H1c: UAA → PSV	0.329	0.062	5.282	0.000	Yes
H2a: ULA → PHV	0.247	0.063	3.921	0.000	No
H2b: ULA → PCV	0.387	0.052	7.425	0.000	Yes
H2c: ULA → PSV	0.141	0.063	2.234	0.026	Yes
H3a: PEA→ PHV	0.300	0.058	5.202	0.000	Yes
H3b: PEA → PCV	0.510	0.046	11.077	0.000	Yes
H3c: PEA→ PSV	0.262	0.053	4.928	0.000	Yes
H4a: PHV → REB	0.254	0.064	3.984	0.000	Yes
H4b: PCV → REB	0.065	0.052	1.260	0.208	No
H4c: PSV → REB	0.521	0.063	8.240	0.000	Yes
Gender → REB	0.083	0.079	1.057	0.290	–
Age → REB	0.120	0.037	3.244	0.001	–
Educational level → REB	−0.063	0.037	1.731	0.084	–
Income → REB	−0.017	0.043	0.399	0.690	–
Number of purchases → REB	0.118	0.045	2.649	0.008	–
Purchase amount → REB	0.101	0.042	2.376	0.018	–

There is a significant positive effect of user activity affordance on perceived hedonic value (*β* = 0.285, *p* < 0.001), and hypothesis H1a is valid. There was no significant effect between user activity affordance and perceived cognitive value (*β* = 0.024, n.s.). User activity affordance significantly and positively influenced perceived social value (*β* = 0.329, *p* < 0.001), hypothesis H1c holds true. User level affordance significantly and positively influenced perceived hedonic value (*β* = 0.247, *p* < 0.001), hypothesis H2a holds. User level affordance cumulatively and significantly positively influenced perceived cognitive value (*β* = 0.387, *p* < 0.001), hypothesis H2b holds. User level affordance positively influenced perceived social value (*β* = 0.141, *p* < 0.05), hypothesis H2c holds. Purchasing special effects affordance positively influenced perceived hedonic value (*β* = 0.300, *p* < 0.001), hypothesis H3a holds. Purchase of special effects affordance significantly and positively influenced perceived cognitive value (*β* = 0.510, *p* < 0.001), hypothesis H3b holds. Purchasing special effects affordance significantly positively affects perceived social value (*β* = 0.262, *p* < 0.001), hypothesis H3c holds. Perceived hedonic value positively influenced repurchasing behavior (*β* = 0.254, *p* < 0.001) and hypothesis H4a was valid. There was no significant positive effect between perceived cognitive value and repurchase behavior (*β* = 0.254, n.s.). Perceived social value positively influenced repurchasing behavior (*β* = 0.521, *p* < 0.001) and hypothesis H4c was established. Gender positively influenced re-purchase behavior (*β* = 0.083, *p* < 0.05), age positively influenced re-purchase behavior (*β* = 0.120, *p* < 0.05), education did not positively influence re-purchase behavior (*β* = −0.063, n.s.), the number of purchases did not positively influence re-purchase behavior (*β* = 0.118, n.s.), and the amount of purchase positively influenced re purchasing behavior (*β* = 0.101, *p* < 0.05).

Finally, this study tested the model’s goodness of fit (GOF) using standardized root mean square residuals (SRMR). The model had an SRMR value of 0.055, which is less than the threshold of 0.08. Therefore, the fit of the model is acceptable (Hair et al., 2017).

### 4.6 Analysis of intermediation effect

There may also be several mediating effects in this study’s model, so the mediating effects were analyzed in this study using PLS-SEM. The results of the analysis are described in Table 5.

**Table 5:** Measurements of path models.

Path	*β*	STDEV	T-value	*p*-value
ULA → PHV → REB	0.063	0.022	2.854	0.004
ULA → PCV → REB	0.025	0.020	1.247	0.212
ULA → PSV → REB	0.073	0.034	2.151	0.032
UAA → PHV → REB	0.072	0.024	2.964	0.003
UAA → PCV → REB	0.002	0.004	0.360	0.719
UAA → PSV → REB	0.171	0.041	4.137	0.000
PEA→PHV → REB	0.076	0.025	3.064	0.002
PEA→PCV → REB	0.033	0.027	1.237	0.216
PEA→PSV → REB	0.137	0.033	4.134	0.000

Between user level affordance and repurchase behavior, perceived hedonic value had a significant mediating effect (*β* = 0.063, *p* < 0.05). Between user level affordance and repurchase behavior, perceived social value had a mediating effect (*β* = 0.073, *p* < 0.05). There was no significant mediating effect of perceived cognitive value between user level affordance and repurchase behavior (*β* = 0.025, n.s.). There is a significant mediating role of perceived hedonic value between user activity affordance and repurchase behavior (*β* = 0.072, *p* < 0.05). There is no significant mediating effect of perceived cognitive value between user activity affordance and repurchase behavior (*β* = 0.002, n.s.). Between user activity affordance and repurchase behavior, perceived social value had a significant mediating effect (*β* = 0.171, *p* < 0.001). There was a significant mediating effect of perceived hedonic value between purchase effects affordance and repurchase behavior (*β* = 0.076, *p* < 0.05). There was no significant mediating effect of perceived cognitive value between purchase effects affordance and repurchase behavior (*β* = 0.0.033, n.s.). There was a significant mediating effect of perceived social value between purchase effects affordance and repurchase behavior. (*β* = 0.137, *p* < 0.001).

## 5 Discussion and conclusions

### 5.1 Key findings

This study aims to analyze, from the perspective of gamification elements, the influencing factors of the re-purchase behavior of game live viewers. Through data collection and analysis, it can be found that user activity affordance has a significant positive impact on perceived hedonic value, which once again verifies the research of Suh et al. (2018), in the game live broadcast, appropriately added to the gamification element of activity, live broadcast viewers can complete the tasks set by the gamification element and get the relevant rewards incentives for the activity, which increases the viewer’s enthusiasm to watch the live broadcast, and completing the tasks will also bring a more interesting experience, which improves the entertainment experience of the audience. This study found that user activity affordance does not have a significant effect on perceived cognitive value, and the possible explanation is that users’ perceptions of live streaming or platforms are generated through the anchor’s live streaming content as well as the platform’s visual design, rather than generating new cognitive value solely due to the affordance of activity. In this study, user activity affordance has a significant positive effect on perceived social value, validating the research of Burke et al. (2011). That is, the higher the user’s activity marker in the live broadcast of the game, the more it increases their exposure, attracts the attention of the host and other fans, improves the chances of making friends with other users, and is a reflection of the user’s perceived social value.

In this study, user level affordance has a significant positive effect with perceived hedonic value, perceived cognitive value, and perceived social value, which validates the study by Sajjadi et al. (2019). By adding the gamification of user level affordance to game live broadcasts, live viewers will receive higher user level ratings or logos and will be more likely to complete tasks and give suggestions when watching game live broadcasts. When live streaming viewers enjoy this gamification, they will be able to better appreciate the fun of live gaming and perceive a stronger hedonistic value. Higher user levels tend to indicate longer online time and live streaming experiences, giving users a deeper gaming awareness. At the same time, higher-level logos will attract the attention of other lower-level fans in the live streaming room, and higher-level users will have more voice, which can improve users’ perception of social value.

Purchase effects affordance has a significant positive effect on perceived hedonic value, perceived cognitive value, and perceived social value, which validates Wang et al. (2024) study. Adding gamification elements related to purchase effects affordance in games can increase users’ consumption opportunities. Users who watch live streams will generate consumption when watching live streams, and the affordance of special effects increases users’ entertainment experience and hedonic value, while the design of special effects allows users to compare with other platforms and have a more in-depth understanding of the perception of live streaming platforms. Increasing the affordance of purchase effects affordance increases the exposure of the user’s account to a greater extent, and the appearance of special effects when giving gifts and tips will attract the attention of the anchor and other water friends and improve the user’s ability to perceive social value.

Perceived hedonic value and perceived social value have a significant positive effect on repurchase behavior, which likewise validates the study of Wang et al. (2022). In game live broadcasting, perceived value will have an impact on viewers’ purchasing behavior in live services, and perceived hedonic value will provide a mediating role. In the process of watching the game live, viewers’ purchasing behavior is the result of balancing the perceived benefits and perceived gains and losses. Viewers spend a certain amount of time and energy to gain pleasure from the live broadcast of the game, the demonstration of the host’s superior skills, and the performance of the live broadcast’s rich content. It is noteworthy that in this study, perceived cognitive value has no significant effect on repurchase behavior. The possible explanation is that in live gaming, viewers repurchase behavior is mainly influenced by the host’s live content or the game itself, whereas users perceived cognitive value only affects users’ willingness to stay on the platform or in the studio to watch the game live at the current moment. It is therefore reasonable to believe that perceived cognitive value may include factors such as understanding game strategies, learning new techniques, or gaining insights from the host. While this can significantly increase viewer engagement and extend their viewing time, it does not necessarily elicit the same emotional triggers (e.g., excitement, admiration, or sense of urgency) that drive repeat purchase behavior. It may also mean that repurchase behavior in live streaming environments is typically more reliant on emotional factors such as connection to the host, emotional resonance with the content, or social signals (e.g., gifting for recognition).

### 5.2 Theoretical contributions

There are some contributions to the theory in this study. First, this study introduces the affordance-psychological outcome-behavior (A-P-B) theory into the study of game live broadcasting, and the analysis of the factors influencing the re-purchase behavior of game live broadcasting viewers expands the scope of the use of the theoretical framework of the affordance-psychological outcome-behavior (A-P-B).

Second, based on the theory of affordance-psychological outcome-behavior (A-P-B), the variable of gamification element affordance and the variable of perceived value are introduced, which confirms that there is a certain influence between gamification element affordance and user’s perceived value in game live broadcasting, and enriches the literature on the business aspects of game live broadcasting as well as consumer behavior. It enriches the literature on the business aspects of game broadcasting and consumer behavior.

Third, the gamification element affordance was downwardly divided into three sub-dimensions: user activity affordance, user level affordance and purchase effects affordance, which enriched the variables of gamification element affordance; and the perceived experiential value was subdivided into three variables: perceived hedonic value, perceived cognitive value and perceived social value, confirming that they have positive effects on repurchase behavior enriching the literature on the commerce aspect of game live broadcasting. Enriched the antecedents of consumer repurchase behavior.

### 5.3 Practical contributions

This study is a contribution to practice and a guide to improving the repurchase behavior of live streaming users. This study found that user activity affordance has a positive impact on perceived hedonic value and perceived social value, so it is possible to increase the logo of user activity in the design of the live broadcasting platform to increase its aesthetics and recognition, and to differentiate active users from diving users, which will increase the live broadcasting viewers’ motivation to watch live broadcasts and interactivity, and it will increase the length of live broadcasting viewing and increase the opportunity to consume. For example, fan leaderboards could be introduced to show top contributors (based on activity or spending) to incentivize engagement; Offer challenges like “watch 3 h of live streams” or “gift 5 subscriptions” to earn rewards. Besides that, platforms can also consider offering more benefits such as ad-free viewing, priority chat access or exclusive content by introducing tiered subscriptions.

Second, this study found that user level affordance has a positive impact on users’ perceived hedonic value, perceived cognitive value and perceived social value. Therefore, it is essential to add user level markings to live broadcasts, and obvious user level markings will improve the user’s viewing experience, increase entertainment and motivation, and increase the likelihood of re-purchase. Many platforms today have adopted the use of visually striking badges next to usernames to indicate levels. Higher levels can have progressively more complex designs, such as animations or glow effects. Time-limited level themes or boosts, such as “Seasonal XP Boosts” that make leveling up more engaging during events, could also be added. In addition, we suggest that gaming live platforms consider allowing premium users to have a say in platform decisions, such as voting on new features or event themes.

Third, this study found that the affordance of purchase effects affordance has a significant positive effect on users perceived hedonic value, perceived cognitive value and perceived social value. Therefore, the inclusion of cool overriding special effects or colorful logos in live broadcasts can significantly improve users’ purchasing experience, thereby increasing the chances of re-purchase by live viewers. Specific approaches include the introduction of visually appealing animated effects related to virtual gifts or purchases that appear prominently during the live stream (e.g., fireworks displays, ribbon themed effects), as well as the suggestion of introducing time-limited effects that users can purchase to stand out on special occasions. We recommend that gaming live platforms consider temporary free trials of certain effects to encourage purchases after the trial ends. Using AI to recommend effects based on user preferences, viewing history or previous purchases is also a good option.

### 5.4 Insufficient research

There are some shortcomings that exist in this study. First of all, this study adopts the snowball survey method, only 232 effective sample size, in general, the sample size is small, may lack representativeness, hope that in the future other research methods can be used to validate the research of this paper, such as big data analysis and so on. Second, this paper only investigates the affordances of activity, level and purchase effects affordances of gamification elements, but in fact, there may be other gamification element affordances in live broadcasting that may have a certain impact on viewers’ re-purchase behaviors, and it is hoped that future research will continue to study them in depth in accordance with the progress of this study. Third, this study only focuses on the Chinese live streaming market, and the extent of reference for live streaming companies in overseas markets is more general, and it is hoped that future research can be conducted for multicultural markets around the world.

## Data Availability

The raw data supporting the conclusions of this article will be made available by the authors, without undue reservation.

## Appendix

**Table:**

Factor	Serial Num.	Item	References
Users activity affordance	UVA1	The game streams I watch regularly provide me with detailed and eye-catching markers of user activity.	Su et al. (2020)
	UVA2	The live gaming streams I often watch can give me an idea of how to boost user activity.	
	UVA3	Live gaming allows me to visualize how active I am.	
User level affordance	ULA1	The game streams I often watch provide me with detailed and eye-catching user level identifiers	Su et al. (2020)
	ULA2	The live gaming streams I often watch allow me to see the attributes of my own user level.	
	ULA3	The live gaming streams I often watch can give me an idea of how to level up users.	
Purchase effect affordance	PEV1	After purchasing a gift, the live game I often watch can provide me with eye-catching special effects	Su et al. (2020)
	PEV2	After purchasing a gift, the special effects available to me from the game streams I often watch can demonstrate the value of the gift	
	PEV3	After purchasing a gift, the special effects provided by the game streams I often watch can give me a good buying experience	
Perceived hedonic value	PVE1	Watching live gaming on my usual platforms, I get so involved that I forget everything!	Nadeem et al. (2021)
	PVE2	Watching live gaming on my usual platforms, I felt a temporary escape from everyday life	
	PVE3	Watching live gaming on my usual platforms is very enjoyable	
	PVE4	I get excited about watching live gaming on my usual platforms	
Perceived cognitive value	PCV1	I think the live gaming service on my usual platform is up to an acceptable standard	Nadeem et al. (2021)
	PCV2	I think the anchors on my usual platform for live gaming are competent and easy to get along with	
	PCV3	I think it’s worth the time and effort spent in live gaming on my usual platforms	
Perceived social value	PSV1	Watching live gameplay on this platform can provide me with recognition within my circle.	Nadeem et al. (2021)
	PSV2	Watching the game live on this platform is important for my in-circle relationships	
	PSV3	Watching the game live on this platform will help me make a good impression on people in my circle	
Repurchase behavior	RB1	I’m going to continue to buy virtual gifts while watching live gaming on this platform, not stop buying them	Wen et al. (2011)
	RB2	If I could, I would continue to buy virtual gifts on live gaming on this platform as much as possible	
	RB3	I’ll probably continue to buy gifts from live gaming streams on this platform in the future, rather than spending money on live gaming streams on other platforms	
